# Optic Disc Change during Childhood Myopic Shift: Comparison between Eyes with an Enlarged Cup-To-Disc Ratio and Childhood Glaucoma Compared to Normal Myopic Eyes

**DOI:** 10.1371/journal.pone.0131781

**Published:** 2015-07-06

**Authors:** Hae-Young Lopilly Park, Sung Eum Kim, Chan Kee Park

**Affiliations:** Department of Ophthalmology and Visual Science, College of Medicine, The Catholic University of Korea, Seoul St. Mary's Hospital, Seoul, Korea; Bascom Palmer Eye Institute, University of Miami School of Medicine;, UNITED STATES

## Abstract

**Background:**

Progressive disc tilting and the development or enlargement of peripapillary atrophy (PPA) are observed during a myopic shift in children. This could be related to the changes around the optic nerve head during eyeball elongation. If the biomechanical properties at or around the optic nerve head are changed after exposure to elevated intraocular pressure (IOP) in glaucoma eyes, different response of the disc tilting and PPA changes could take place during eyeball elongation by myopic shift. On the basis of this background, the aim of this study was to compare the morphological changes in the optic disc induced by a myopic shift during childhood between normal control eyes, eyes from disc suspects with an enlarged cup-to-disc ratio (CDR), and eyes with childhood glaucoma.

**Methods:**

Total of 82 eyes from 82 subjects younger than 14 years of age were included in the study. Serial disc photographs were classified into one of two groups: eyes with an optic nerve head (ONH) or peripapillary atrophy (PPA) change or without an ONH/PPA change. Using ImageJ software, the outlines of the optic disc and PPA were plotted, and the vertical disc diameter (VDD), horizontal disc diameter (HDD), and maximum PPA width (PPW) were measured. The changes in the ratios of these parameters and the relationships between the degree of myopic shift or the ONH/PPA change were analyzed.

**Results:**

Twenty-five eyes with normal optic disc appearance, 36 eyes with enlarged cup-to-disc ratio, and 21 eyes of glaucoma patients were analyzed. The initial intraocular pressure (IOP) at diagnosis was significantly different among the groups (*P*<0.001). The degree of myopic shift during follow-up period was not significantly different among the groups (*P*=0.612). However, the changes in the HDD/VDD and PPW/VDD ratios were significantly greater in the disc suspect group and significantly smaller in the glaucoma group. Among the 42 eyes with an ONH/PPA change, 16 (38.1%) were from the normal control group, 24 (57.1%) were from the disc suspect group, and 2 (4.8%) were from the glaucoma group (*P* < 0.001).

**Conclusions and Relevance:**

The optic disc change during childhood myopic shift was different in eyes with various conditions. Eyes of childhood glaucoma showed less change in the disc morphology during myopic shift compared to eyes with normal disc or enlarged cup-to-disc ratio.

## Introduction

The reversal of optic disc cupping and a change in the disc area after surgical intraocular pressure (IOP) reduction is commonly found in patients with childhood glaucoma, especially in patients in an early stage of primary congenital glaucoma (PCG).[1–3] These changes in the optic disc have been reported to be related to the percentage change in IOP, the axial length, and the age at surgery.[1, 2] This may be related to the biomechanical properties of the lamina cribrosa and peripapillary sclera, which vary depending on age and axial length. Clinically obvious cupping reversal is less frequently observed in adults after surgery, which may reflect a decrease in the elasticity of the lamina cribrosa and the peripapillary sclera with aging. Likewise, chronic IOP elevation and glaucoma are also thought to change the biomechanical properties of the lamina cribrosa and the peripapillary sclera.[4–7] Our group has reported that changes in the optic nerve head (ONH) in eyes with glaucoma were different depending on the duration of the IOP elevation, which indirectly suggests that chronically elevated IOP may stiffen the ONH structure.[8]

A recent study by Kim et al. documented progressive disc tilting and the development or enlargement of peripapillary atrophy (PPA) during a myopic shift in children with a suspected glaucomatous optic disc.[9] Because axial elongation occurs during the development of myopia, predominantly posterior scleral thinning and expansion of the posterior eyeball, including the ONH may induce morphological changes in the optic disc.[10, 11] In contrast, we observed that the optic disc in eyes of PCG or juvenile open-angle glaucoma (JOAG) patients seem to display fewer morphological changes caused by the myopic shift. The eyes of PCG or JOAG subjects were exposed to elevated IOP, which may have altered the biomechanical properties of the lamina cribrosa and the peripapillary sclera. On the basis of this observation, the aim of this study was to compare the morphological changes in the optic disc induced by a myopic shift during childhood between normal control eyes, eyes from disc suspects with an enlarged cup-to-disc ratio (CDR), and eyes with childhood glaucoma.

## Methods

### Subjects

Subjects younger than 14 years of age who developed myopia with serial optic disc photographs were enrolled in the study. Subjects were consecutively enrolled from a database of patients examined at the glaucoma clinic of Seoul St. Mary’s Hospital. Medical records of disc suspects with enlarged CDR and patients with childhood glaucoma were reviewed between January 2002 and December 2014. Normal controls were enrolled prospectively in the pediatric clinic between January 2009 and May 2013. The Institutional Review Board of Seoul St. Mary’s Hospital approved this study, which adhered to the principles of the Declaration of Helsinki. Written informed consent was obtained from the next of kin, or guardians on behalf of the children.

Each subject received a complete ophthalmic examination, including a medical history review, the measurement of best-corrected visual acuity, cycloplegic refraction measurement with automated refractometer, slit-lamp biomicroscopy, Goldmann applanation tonometry or tonopen measurements, gonioscopy, central corneal thickness measurement using ultrasound pachymetry (Tomey Corporation, Nagoya, Japan), and disc photography (Kowa VX-10, Nagoya, Japan). For inclusion in the study, subjects were required to have at least two optic disc photographs with an interval of at least 1 year. After detailed ophthalmic assessment, the right eye was randomly chosen for inclusion in cases which both eyes of the patient were eligible for the study.

The subjects were classified into the following groups: normal controls, disc suspects with an enlarged CDR, and glaucoma including PCG and JOAG. The diagnosis of PCG was established via a documented elevated IOP (> 21 mmHg), enlarged corneal diameter (> 12.5 mm), and an abnormal CDR (CDR > 0.4) in the first year of life. The diagnosis of JOAG was made according to the following criteria: age below 14 years; elevated IOP (> 21 mmHg); and glaucomatous optic disc or retinal nerve fiber layer (RNFL) changes (such as diffuse or localized rim thinning, a notch in the rim, enlarged vertical cupping, or RNFL thinning) from red-free photograph, confirmed by two glaucoma specialists (HYP, CKP); and an open angle on the gonioscopic examination. The enlarged CDR group included eyes with an IOP of ≤ 21 mmHg in repeated measurements, and a normal optic disc (no rim thinning or loss, no RNFL thinning) except an enlarged CDR > 0.4. The normal control group comprised subjects with an IOP of ≤ 21 mmHg on repeated measurements and normal optic disc and RNFL (CDR <0.4, no asymmetry of CDR, no rim thinning or loss, no RNFL thinning).

The following exclusion criteria were implemented: a spherical equivalent (SE) more than -6 diopters (D) at initial examination; significant systemic illness; significant ocular diseases other than glaucoma; Axenfeld–Rieger’s anomaly; Peters’ anomaly; previous uveitis; previous trauma; history of retinopathy of prematurity; previous corneal, cataract, or retinal surgery; corneal opacification or visible corneal edema; nystagmus; or poor compliance.

### Disc Photography

The disc photographs were acquired using a digital camera (Kowa VX-10, Nagoya, Japan) after maximal pupil dilation. The optic disc images were independently evaluated by two observers (HYP and HY) who were blinded to each other and to the patient’s information. The method of measurement and classification of ONH/PPA changes were from a study by Kim et al.[9]

Each observer classified the photographs into one of two groups: eyes with an ONH/PPA change or eyes without an ONH/PPA change. Any discrepancy between the observers was resolved by consensus including the opinion of a third observer (CKP). The final disc photograph was overlaid onto the baseline disc photograph, and the blood vessel contour around the ONH served as a reference. Only those photographs taken with a similar angle of viewing were included in the analysis. The location of the disc margin relative to the blood vessels around the ONH and the presence of PPA were used to determine the presence of any changes in the ONH and PPA.

The serial disc photographs were evaluated by one observer (HYP) who was blinded to the patients’ information. The outlines of the optic disc (the inner border of Elschnig’s scleral ring) and PPA (an inner crescent of chorioretinal atrophy with visible sclera and choroidal vessels) were plotted using ImageJ software (available at http://rsb.info.nih.gov/ij/index.html). The vertical disc diameter (VDD), horizontal disc diameter (HDD), and maximum PPA width (PPW) were then measured from the baseline and final disc photographs. The ratios of HDD to VDD (HDD/VDD) and PPW to VDD (PPW/VDD) were calculated from the measurements. To evaluate the interobserver reproducibility of our measurements, 20 randomly selected disc photographs were assessed by two examiners.

### Statistical analysis

The interobserver reproducibility of the measurements of the optic disc and PPA parameters were evaluated by calculating intraclass correlation coefficients.

The Kruskal-Wallis one-way ANOVA test was used to compare the differences among the groups. Comparisons between two groups were performed using Student’s *t*-test or the Mann-Whitney U test. The *chi*-square test was used, where appropriate, to compare frequencies. To identify the factors associated with the ONH/PPA changes, univariate and multivariate logistic regression analyses were performed. The dependent variable was the presence of ONH/PPA changes. The independent variables were patient age, gender, diagnosis, baseline SE, SE changes, follow-up period, central corneal thickness, and IOP. The variables exhibiting significance values of *P* < 0.10 upon univariate analysis were included in the multivariate model. Correlation studies were performed using linear regression. A *P* value of less than 0.05 was considered to be statistically significant. Statistical analysis was performed using the SPSS statistical package (SPSS, Chicago, IL, USA).

## Results

A total of 82 eyes from 82 subjects younger than 14 years of age who met the inclusion and exclusion criteria were included in the study. Out of the 82 eyes, 25 were normal controls, 36 were disc suspects, and 21 were eyes with glaucoma.

The youngest mean age at the time of the initial disc photograph examination was in the glaucoma group (2.52 ± 3.04 years; range, 2–12), followed by the normal control (5.12 ± 2.84 years; range, 1–14), and the disc suspect (5.34 ± 2.25 years; range, 3–14) groups. The mean age at the time of the initial disc photograph examinations was significantly different among the groups (*P* < 0.001). The mean follow-up period was the longest for the glaucoma group (5.73 ± 3.29 years), followed by the normal control (2.60 ± 1.96 years), and the disc suspect (2.55 ± 1.59 years) groups. The baseline mean SE was -1.98 ± 1.74 D (range, -5.13 to +0.25 D) for the normal control group, -1.62 ± 1.55 D (range, -4.88 to +0.25 D) for the disc suspect group, and -1.78 ± 1.61 D (range, -4.63 to -0.25 D) for the glaucoma group. The baseline mean SE was not significantly different among the groups (*P* = 0.107). The final mean SE was significantly different among the groups (*P* = 0.006), with the normal control group developing significant myopia (-3.56 ± 1.39 D) compared to the other groups. The degree of myopic shift was not significantly different among the groups (*P* = 0.612). The mean myopic shift was the greatest for the disc suspect (-1.64 ± 1.50 D) group, followed by the normal control (-1.57 ± 1.15 D), and the glaucoma (-1.23 ± 1.29 D) groups. The initial IOP at diagnosis was significantly different among the groups (*P* < 0.001; Table 1).

**Table 1:** Characteristics of childhood subjects with myopic shift.

	Normal controls	Disc suspects	Glaucoma	*P* Value	Multiple comparison^‡^
	(n = 25)	(n = 36)	(n = 21)		
Age at baseline, year	5.12 ± 2.84	5.34 ± 2.25	2.52 ± 3.04	<0.001*	D = N > G
Age at final follow-up, year	7.78 ± 2.56	7.82 ± 1.96	8.16 ± 3.25	0.426*	
Gender, Male:Female	11:14	16:20	10:11	0.851^†^	
Duration between baseline and final follow-up, years	2.60 ± 1.96	2.55 ± 1.59	5.73 ± 3.29	<0.001*	G > N = D
Sperical equivalent, diopter					
Baseline	-1.98 ± 1.74	-1.62 ± 1.55	-1.78 ± 1.61	0.107*	
Final	-3.56 ± 1.39	-3.26 ± 1.89	-2.89 ± 1.73	0.006*	D = G < N
Changes	-1.57 ± 1.15	-1.64 ± 1.50	-1.23 ± 1.29	0.612*	
Central corneal thickness, μm	596.67 ± 29.50	546.33 ± 21.90	573.20 ± 46.47	0.139*	
Intraocular pressure, mmHg					
Baseline	15.25 ± 2.11	16.02 ± 6.20	29.58 ± 3.22	<0.001*	G > D = N
Mean during follow-up	15.81 ± 2.22	15.64 ± 3.15	17.85 ± 3.05	0.565*	G = D = N

Data are presented as mean ± standard deviation.

*Comparison among the three groups by Kruskal-Wallis One-way analysis of variance.

^†^Comparison among the three groups by Chi-square test.

^‡^Comparison between the two groups by Mann-Whitney U test.

Table 2 displays the ONH and PPA characteristics at baseline and in the final follow-up period. There was an excellent interobserver reproducibility for the measurement of the HDD/VDD and PPW/VDD ratios (intraclass correlation coefficients = 0.963 and 0.947, respectively). The HDD/VDD was not different among the groups at baseline (*P* = 0.328) or at the final examination (*P* = 0.147). The change in the HDD/VDD ratio was also not different among the groups (*P* = 0.187); however, multiple comparisons revealed that there was a significantly smaller change in the glaucoma group. The PPW/VDD ratio was not different among the groups at baseline (P = 0.243). However, the PPW/VDD ratio at the final examination (*P* = 0.016) and the changes (*P* = 0.033) were significantly different among groups. Multiple comparison analysis revealed that the glaucoma group had the smallest PPW/VDD ratio both at baseline and at the final examination. The analysis of the PPW/VDD ratio indicated a significantly smaller change in the glaucoma group.

**Table 2:** Optic disc morphological changes in eyes with normal optic disc, disc suspects with enlarged cup-to-disc ratio, and glaucoma.

	Normal controls	Disc suspects	Glaucoma	*P* Value	Multiple comparison^‡^
	(n = 25)	(n = 36)	(n = 21)		
Horizontal disc diameter to vertical disc diameter ratio					
Baseline	0.919 ± 0.122	0.880 ± 0.103	0.931 ± 0.096	0.328*	
Final	0.882 ± 0.112	0.841 ± 0.120	0.922 ± 0.088	0.147*	G > D = N
Changes	-0.027 ± 0.072	-0.038 ± 0.075	-0.008 ± 0.040	0.018*	D > N > G
Maximum peripapillary width to vertical disc diameter ratio					
Baseline	0.100 ± 0.132	0.109 ± 0.125	0.053 ± 0.070	0.243*	D = N > G
Final	0.138 ± 0.100	0.179 ± 0.155	0.074 ± 0.091	0.016*	D = N > G
Changes	0.040 ± 0.073	0.069 ± 0.155	0.021 ± 0.046	0.033*	D = N > G

Data are presented as mean ± standard deviation.

*Comparison among the three groups by One-way analysis of variance.

^†^Comparison between the two groups by Mann-Whitney U test.

Out of the 82 eyes, 42 (51.2%) were classified into the ONH/PPA change group and 40 (48.8%) were classified into the ONH/PPA unchanged group (Table 3). The two groups were not different regarding the baseline age or follow-up periods. The baseline (*P* = 0.094) and final mean SE (*P* < 0.001) were more myopic in the ONH/PPA change group compared to the ONH/PPA unchanged group. The mean myopic shift was greater in the ONH/PPA change group (-1.85 ± 1.36 D) compared to the ONH/PPA unchanged group (-1.02 ± 1.06 D, *P* = 0.004). The HDD/VDD ratio was smaller and the PPW/VDD ratio was larger at baseline and at the final examination in the ONH/PPA change group. The mean changes in the HDD/VDD and the PPW/VDD ratios were also significantly greater in the ONH/PPA change group. Among the 42 eyes with an ONH/PPA change, 16 (38.1%) were from the normal control group, 24 (57.1%) were from the disc suspect group, and 2 (4.8%) were from the glaucoma group. These data indicate that there is a significant difference in the distribution of eyes with an ONH/PPA change among the groups (*P* < 0.001; Table 4).

**Table 3:** Comparison between childhood subjects with or without optic disc morphological changes.

	ONH/PPA unchanged group	ONH/PPA change group	*P* Value
	(n = 40)	(n = 42)	
Age at baseline, year	5.47 ± 4.08	4.84 ± 3.50	0.472*
Gender, Male:Female	18:22	19:23	0.983^†^
Duration between baseline and final follow-up, years	3.47 ± 3.08	2.84 ± 1.96	0.290*
Sperical equivalent, diopter			
Baseline	-1.84 ± 1.64	-2.54 ± 1.89	0.094*
Final	-2.87 ± 1.65	-4.37 ± 1.74	<0.001*
Changes	-1.02 ± 1.06	-1.85 ± 1.36	0.004*
Central corneal thickness, μm Intraocular pressure, mmHg	562.88 ± 40.84	564.60 ± 27.40	0.907*
Baseline	23.34 ± 3.24	15.73 ± 2.19	<0.001*
Mean during follow-up	16.48 ± 3.41	16.38 ± 2.79	0.884*
Horizontal disc diameter to vertical disc diameter ratio			
Baseline	0.911 ± 0.104	0.891 ± 0.107	0.422*
Final	0.921 ± 0.103	0.831 ± 0.111	0.001*
Changes	0.009 ± 0.037	-0.060 ± 0.069	<0.001*
Maximum peripapillary width to vertical disc diameter ratio			
Baseline	0.075 ± 0.116	0.107 ± 0.115	0.227*
Final	0.069 ± 0.096	0.212 ± 0.119	<0.001*
Changes	-0.006 ± 0.045	0.104 ± 0.057	<0.001*

Data are presented as mean ± standard deviation.

ONH = optic nerve head; PPA = peripapillary atrophy.

*Comparison among the three groups by Student’s *t*-test.

^†^Comparison among the three groups by Chi-square test.

**Table 4:** The distribution of childhood subjects with or without optic disc morphological changes in eyes with normal optic disc, disc suspects with enlarged cup-to-disc ratio, ocular hypertension, and glaucoma.

	Normal controls	Disc suspects	Glaucoma	*P* Value*
	(n = 25)	(n = 36)	(n = 21)	
ONH/PPA unchanged group	9 (22.5%)	12 (30.0%)	19 (47.5%)	<0.001
ONH/PPA change group	16 (38.1%)	24 (57.1%)	2 (4.8%)	

Data are presented as number (%).

*Comparison among the three groups by Chi-square test.

Logistic regression analysis was performed to determine which factors are related to an ONH/PPA change (Table 5). Based on the univariate logistic regression analysis, patient age, diagnosis, baseline SE, change in the SE, and IOP were all factors related to a change in the ONH/PPA. Based on the multivariate logistic regression analysis, the diagnosis, SE change, and IOP were all factors related to the ONH/PPA change.

**Table 5:** Related factors to optic disc morphological changes in childhood subjects with myopic shift.

	Univariate analysis			Multivariate analysis		
	OR	95% CI	*P* Value	OR	95% CI	*P* Value
Age	0.722	0.680–1.026	0.051	0.784	0.641–0.920	0.082
Gender (male)	0.436	0.190–1.003	0.065	0.596	0.061–5.875	0.658
Diagnosis						<0.001
Normal controls	ref					
Disc suspect	16.889	3.178–89.742	0.001	22.628	3.024–139.312	0.002
Glaucoma	0.917	0.809–0.998	0.031	4.630	1.424–50.557	0.049
Baseline SE	0.817	0.680–0.983	0.032	1.025	0.588–1.785	0.932
SE change	0.635	0.456–0.884	0.007	0.467	0.205–1.066	0.043
Duration between baseline and final follow-up periods	0.864	0.723–1.032	0.107			
Central corneal thickness	1.004	0.987–1.020	0.670			
Mean IOP during follow-up	0.989	0.861–1.138	0.989			

SE = spherical equivalent; IOP = intraocular pressure; OR = odds ratio; CI = confidence intervals.

Logistic regression analysis with dependent variable as the presence of optic nerve head/peripapillary atrophy changes.

Variables exhibiting significance values of *P* < 0.10 upon univariate analysis were included in the multivariate model.

**Fig 1:**
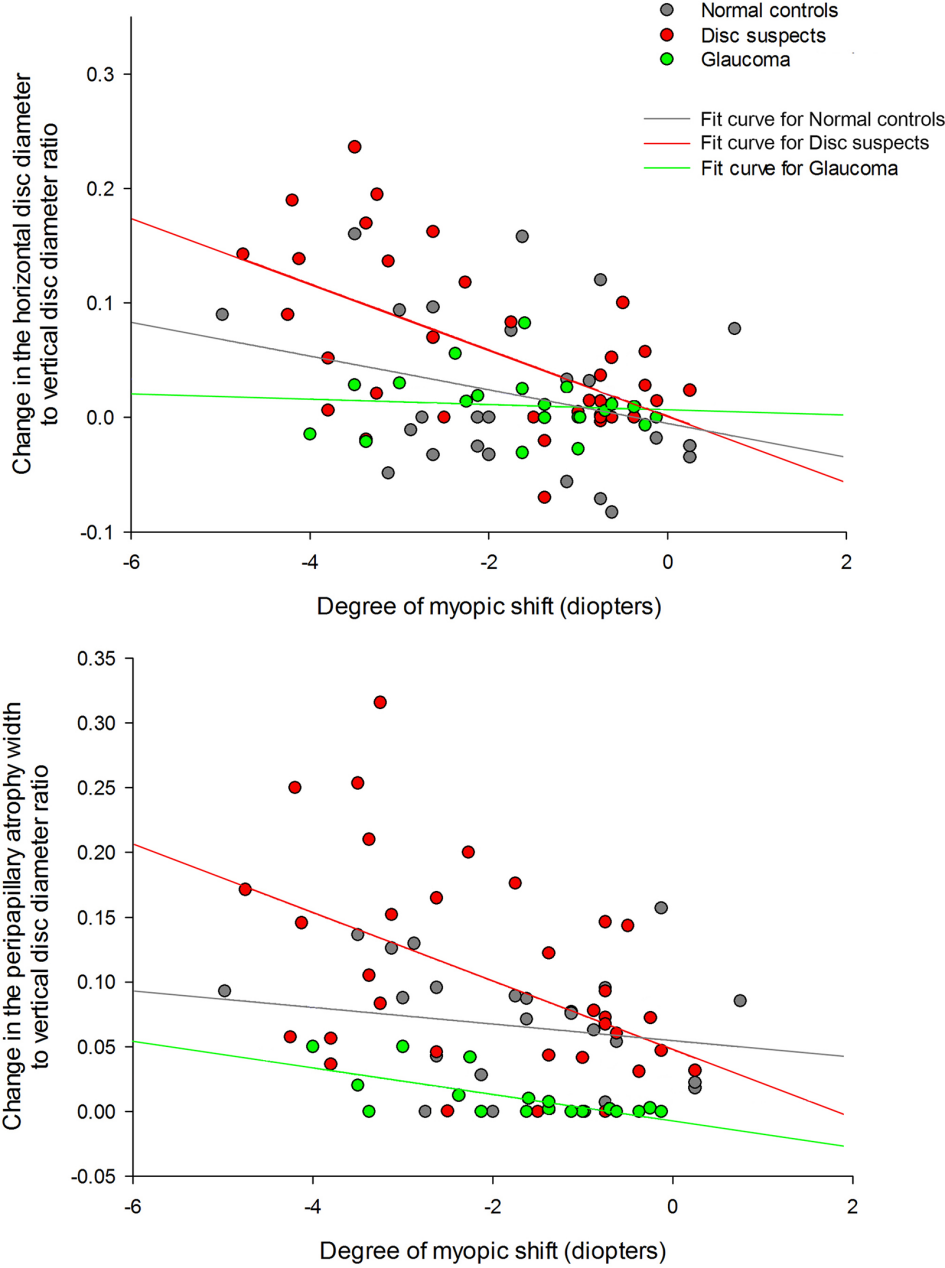
The relationship between the degree of myopic shift and the changes in optic disc morphology. The horizontal disc diameter to vertical disc diameter ratio (upper panel) and the peripapillary atrophy width to vertical disc diameter ratio (lower panel) significantly changed according to the degree of myopic shift in the disc suspect eyes with an enlarged cup-to-disc ratio. The eyes with glaucoma had minimal changes in the two parameter ratios despite the myopic shift.

The change in the HDD/VDD (*r* = -0.450, *P* = 0.006) or the PPW/VDD (*r* = -0.446, *P* = 0.006) ratios significantly correlated with the degree of the SE change only in the disc suspect group (Fig 1).

## Discussion

We compared the optic disc change that occurs with myopia during childhood under various conditions. The changes in the SE during the follow-up period were similar between the normal control eyes, disc suspects, and childhood glaucoma eyes. However, changes in the optic disc ovality and the development of or increases in PPA were significantly greater in the disc suspect eyes and significantly less in the eyes with childhood glaucoma. Factors that were related to the ONH/PPA change included the diagnosis and the amount of SE change.

**Fig 2:**
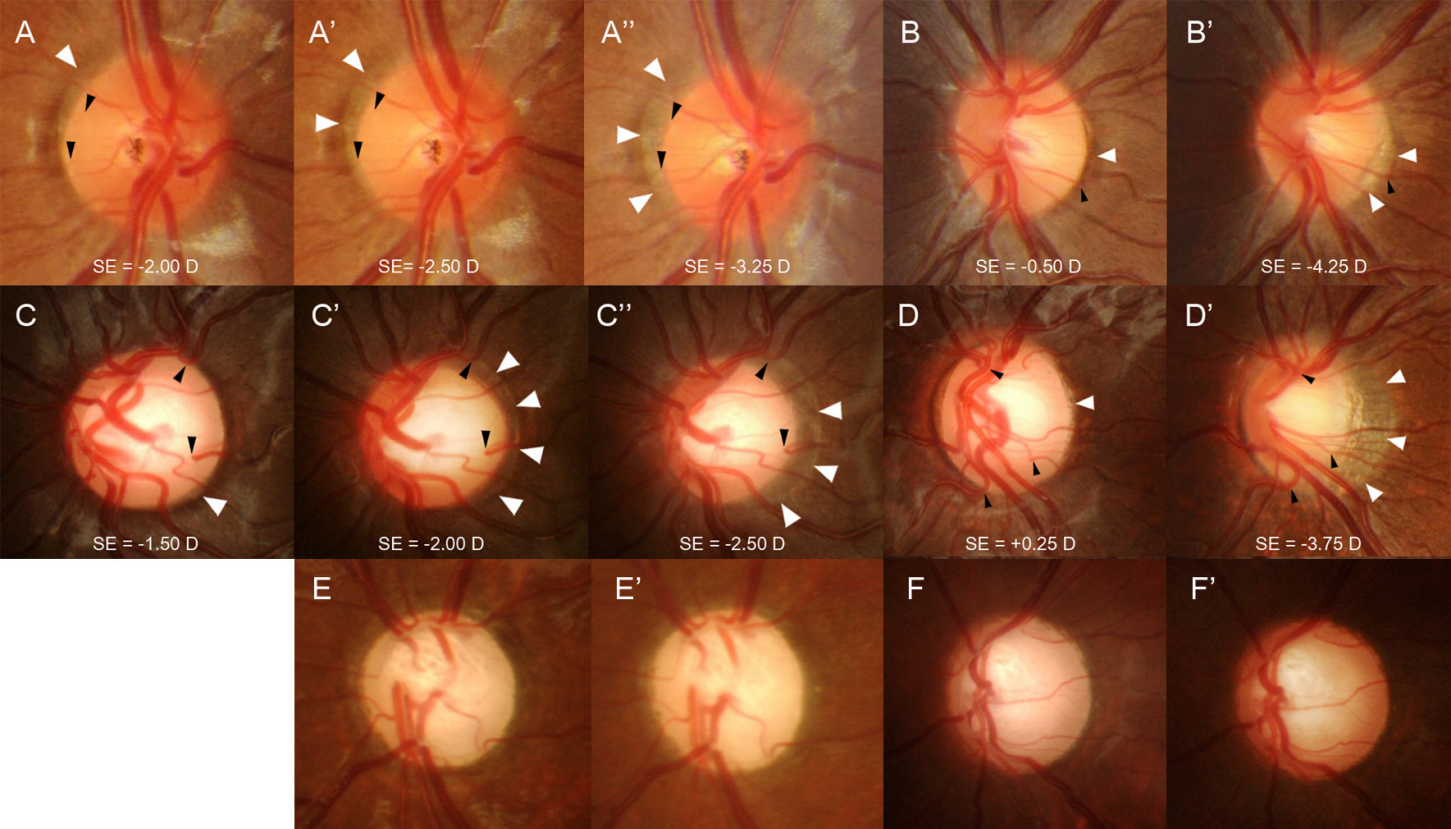
Six cases of childhood eyes with a myopic shift. Eyes with a normal optic disc (A and B) and eyes with an enlarged cup-to-disc ratio (CDR) (C and D) that have progressive disc tilting and development/enlargement of peripapillary atrophy (PPA). The previous disc margin is the PPA margin in follow-up photographs (white arrowheads). Note that the cilioretinal vessels (black arrowheads) at the disc margin move into the PPA region as the disc tilt progresses. Serial disc photographs show that with a similar myopic shift, approximately 1.00 to 1.25 diopters by refraction, the eye with an enlarged CDR (C, C’, and C”) had more prominent disc tilting and development/enlargement of PPA compared to the eye with a normal optic disc (A, A’, and A”). In eyes with a larger myopic shift, approximately 3.75 to 4.00 diopters by refraction, progressive disc tilting and development/enlargement of PPA was greater in the eyes with an enlarged CDR (D and D’) compared to the eyes with a normal disc (B and B’). Eyes with primary congenital glaucoma (E and F) had nearly no disc tilting or development of peripapillary atrophy during the myopic shift of approximately 1.75 to 3.50 diopters by refraction.

Progressive changes in disc tilting and the development/enlargement of PPA in myopic eyes have been suggest that the disc shape change is due to scleral stretching associated with the axial elongation of the eyeball.[9] The eyes from the normal control and disc suspect groups had a similar mean age but were significantly different regarding changes in the ONH/PPA. The eyes with childhood glaucoma were the youngest in age but had a significantly smaller ONH/PPA change compared to the other groups. The present study demonstrates that it is possible that the properties of the sclera may determine the amount of disc shape change that is associated with the development of myopia. As shown the in representative cases, compared to normal control eyes (Fig 2A and 2B), the eyes with an enlarged CDR (Fig 2C and 2D) had more pronounced disc shape changes and displayed the development or enlargement of PPA even with a similar myopic shift in refractions. In contrast, the eyes with glaucoma (Fig 2E and 2F) had only minimal changes in the disc shape after the myopic shift in refraction. To our knowledge, this is the first study that examined and compared the ONH changes between various subgroups, glaucomatous eyes in particular.

Recently, investigations of human cadaver eyes revealed a difference in the biomechanical properties of the sclera of glaucomatous and non-glaucomatous eyes.[12–14] The biomechanical characteristics of the peripapillary sclera produced significant changes in the scleral canal expansion and lamina cribrosa deformation in inflation studies.[15] Corneal hysteresis detected with the Ocular Response Analyzer may be another *in vivo* measurement that indirectly reflects the stiffness of the ONH complex.[16] Corneal hysteresis has been reported to be reduced in eyes with glaucoma.[16, 17] A high IOP, in particular, greatly influences corneal hysteresis compared to various other factors; a negative relationship between corneal hysteresis and IOP was found only in the glaucoma and ocular hypertension groups.[18–20] These findings may represent altered ocular tissue biomechanics in eyes with glaucoma and eyes exposed to elevated IOP. Eyes with glaucoma had minimal optic disc changes that were induced by a myopic shift. This may be a clinical observation that represents stiffened ONH complex or character of the sclera resulting from glaucoma or elevated IOP. However, this is just our hypothesis in interpreting the findings of our study. This study demonstrated that there is difference in the ONH response to axial elongation in eyes of various subgroups, which may be an important finding that should be further investigated.

Some important points should be address in interpreting our results. A previous study reported that a greater myopic shift and an age between 7 and 9 years were factors related to the ONH/PPA change.^9^ In our study, the mean age of the patients from normal control and disc suspect groups had a similar mean age but was significantly different regarding changes in the ONH/PPA. The eyes with childhood glaucoma were the youngest in age but had a significantly smaller ONH/PPA change compared to the other groups. In glaucoma eyes, baseline high IOP at young age may have already induced ONH/PPA changes showing lesser change during the study observation period. Or when IOP was reduced, the changes of ONH/PPA may have recovered in some degree. If disc photographs before glaucoma surgery or at the onset of disease were included, we may have observed the ‘true’ changes, however, this was only possible in some patients. Age at observation was younger in the glaucoma group and there are possibilities that the observation was made before the age when ONH/PPA change may have occurred. However, the final follow-up age was not different between three groups.

Our study has several limitations that must be acknowledged. First, it had a relatively small sample size and the follow-up period was relatively short. Second, it is difficult to generalize our findings because all of the subjects were referred to a tertiary hospital and only Korean individuals were included. We could only assume axial elongation according to SE changes because most subjects did not have information regarding axial length. However, it is known that the myopic shift occurring in children is mostly due to axial elongation.[9, 21] Further investigation is required to confirm the relationship between axial length and optic disc changes. Ocular minification or magnification could not be considered because information on the axial length was lacking. Therefore, we only used parameters defined by ratios, because the parameter ratios are not affected by these errors.

In conclusion, we observed optic disc changes during the childhood myopic shift were different among eyes from normal controls, disc suspects with an enlarged CDR, and glaucoma eyes. This observation may enhance our understanding regarding the differences in optic disc morphology between individuals and the resulting clinical implications.
