# Correction: Optic Disc Change during Childhood Myopic Shift: Comparison between Eyes with an Enlarged Cup-To-Disc Ratio and Childhood Glaucoma Compared to Normal Myopic Eyes

**DOI:** 10.1371/journal.pone.0137938

**Published:** 2015-09-03

**Authors:** Hae-Young Lopilly Park, Sung Eum Kim, Chan Kee Park

There are errors in Fig 2, “Six cases of childhood eyes with a myopic shift.” Please see the corrected Fig 2 here.

**Fig 2:**
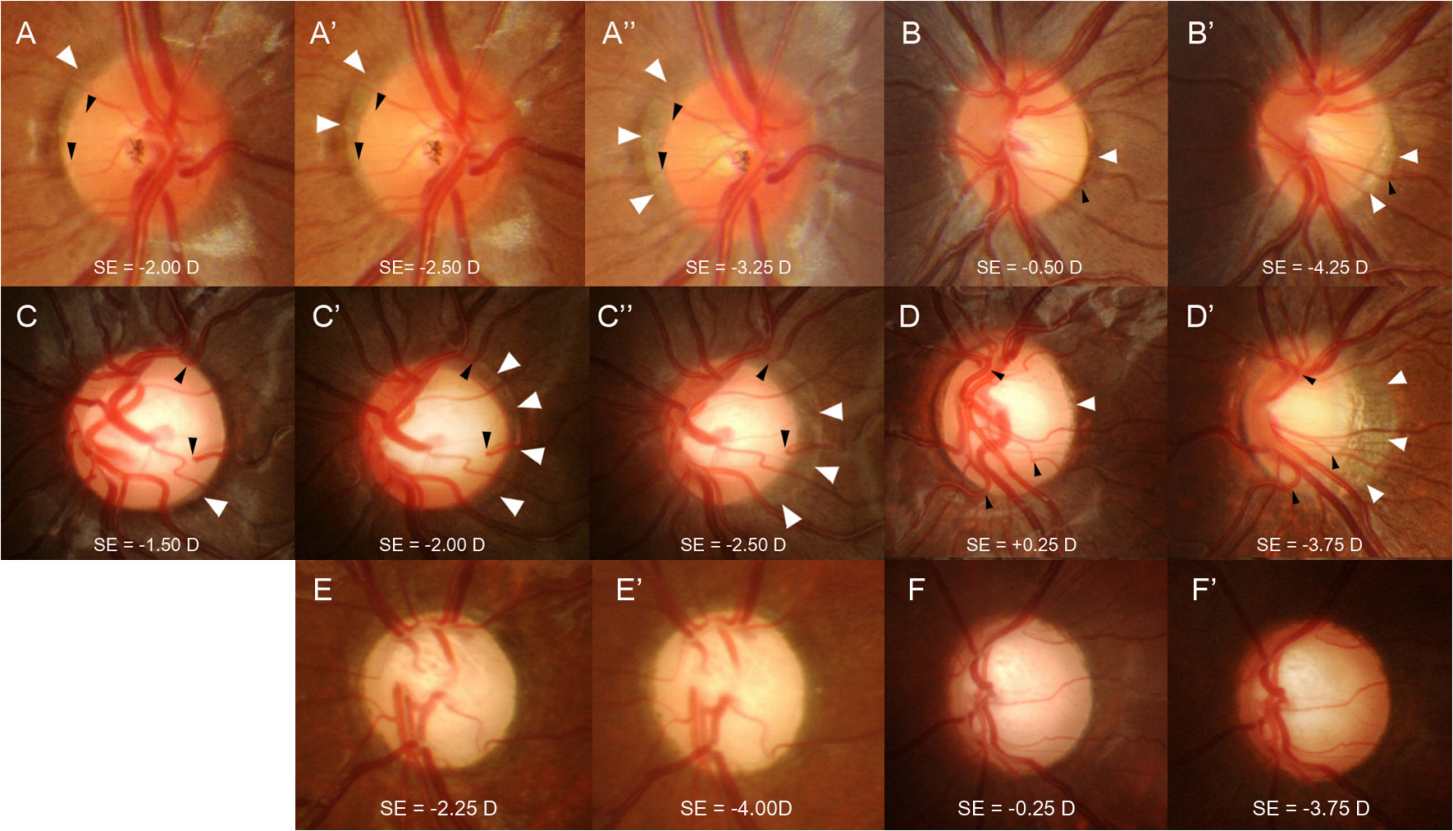
Six cases of childhood eyes with a myopic shift. Eyes with a normal optic disc (A and B) and eyes with an enlarged cup-to-disc ratio (CDR) (C and D) that have progressive disc tilting and development/enlargement of peripapillary atrophy (PPA). The previous disc margin is the PPA margin in follow-up photographs (white arrowheads). Note that the cilioretinal vessels (black arrowheads) at the disc margin move into the PPA region as the disc tilt progresses. Serial disc photographs show that with a similar myopic shift, approximately 1.00 to 1.25 diopters by refraction, the eye with an enlarged CDR (C, C’, and C”) had more prominent disc tilting and development/enlargement of PPA compared to the eye with a normal optic disc (A, A’, and A”). In eyes with a larger myopic shift, approximately 3.75 to 4.00 diopters by refraction, progressive disc tilting and development/enlargement of PPA was greater in the eyes with an enlarged CDR (D and D’) compared to the eyes with a normal disc (B and B’). Eyes with primary congenital glaucoma (E and F) had nearly no disc tilting or development of peripapillary atrophy during the myopic shift of approximately 1.75 to 3.50 diopters by refraction.
