# Correction: Optic Disc Change during Childhood Myopic Shift: Comparison between Eyes with an Enlarged Cup-To-Disc Ratio and Childhood Glaucoma Compared to Normal Myopic Eyes

**DOI:** 10.1371/journal.pone.0150316

**Published:** 2016-02-22

**Authors:** Hae-Young Lopilly Park, Sung Eun Kim, Chan Kee Park

The second author’s name is spelled incorrectly in the original article and in the correction published on September 3, 2015. The correct name is: Sung Eun Kim.
